# Habitual Dietary Patterns, Nutrient Intakes, and Adherence to the Mediterranean Diet among New Zealand Adults: The NZ MED Cross-Sectional Study

**DOI:** 10.3390/nu15122663

**Published:** 2023-06-07

**Authors:** Amy L. Lovell, Rajshri Roy, Alana Klein, Alana Cavadino, Meika Foster, Jeremy D. Krebs, Andrea Braakhuis, Troy L. Merry

**Affiliations:** 1 Department of Nutrition and Dietetics, Faculty of Medical and Health Sciences, The University of Auckland, Auckland 1023, New Zealand; r.roy@auckland.ac.nz (R.R.); akle100@aucklanduni.ac.nz (A.K.); a.braakhuis@auckland.ac.nz (A.B.); t.merry@auckland.ac.nz (T.L.M.); 2 High-Value Nutrition Ko Ngā Kai Whai Painga National Science Challenge, The Liggins Institute, Auckland 1023, New Zealandjeremy.krebs@otago.ac.nz (J.D.K.); 3 Edible Research Ltd., Canterbury 7692, New Zealand; 4 Department of Medicine, University of Otago, Wellington 6242, New Zealand

**Keywords:** Mediterranean Diet, dietary patterns, obesity, cardiovascular disease, diabetes, New Zealand, diet quality’ nutrition’ food frequency questionnaire

## Abstract

There is increasing evidence that adherence to a Mediterranean dietary pattern reduces the incidence of diet-related diseases. To date, the habitual dietary intake of New Zealand (NZ) adults has not been examined in relation to its alignment with a Mediterranean-style dietary pattern. This study aimed to define the habitual dietary patterns, nutrient intakes, and adherence to the Mediterranean Diet in a sample of 1012 NZ adults (86% female, mean age 48 ± 16 years) who had their diabetes risk defined by the Australian Type 2 Diabetes Risk Assessment Tool (AUSDRISK). Dietary intakes were collected using a validated semi-quantitative NZ food frequency questionnaire, and dietary patterns were identified using principal component analysis. Reported intakes from the FFQ were used in conjunction with the Mediterranean-Style Dietary Pattern Score (MSDPS) to determine adherence to a Mediterranean dietary pattern. Mixed linear models were used to analyze the association between dietary patterns and MSDPS with demographics, health factors, and nutrient intakes. Two distinct dietary patterns were identified: Discretionary (positive loadings on processed meat, meat/poultry, fast food, sweet drinks, and sugar, sweets, and baked good) and Guideline (positive loadings on vegetables, eggs/beans, and fruits). Adherence to dietary patterns and diet quality was associated with age and ethnicity. Dietary patterns were also associated with sex. Adherence to a Mediterranean dietary pattern defined by the MSDPS was low, indicating that a significant shift in food choices will be required if the Mediterranean Diet is to be adopted in the NZ population.

## 1. Introduction

The Mediterranean Diet originated from the abundant supply of olives and plant foods in low-income, rural communities in the Mediterranean Basin [1,2]. The Mediterranean dietary pattern is characterized by a high consumption of plant-based foods, a low consumption of red meat and other processed foods, the use of olive oil as the main source of fat, and a moderate intake of wine during meals [1,2,3]. Evidence that accumulated over the past decade has shown that the Mediterranean Diet confers substantial health benefits in the prevention and control of chronic diseases, including cardiometabolic disease [4]. However, a limited number of studies have explored the translation of a Mediterranean Diet to non-Mediterranean countries [5,6,7,8,9]. No studies have measured the degree to which New Zealanders are following this dietary pattern in free living adults 18–70 years of age.

There are differences between the recommendations in the Eating and Activity Guidelines for NZ Adults [10] and the diet and lifestyle recommendations of the Mediterranean Diet pyramid [11,12]. While both dietary patterns include all the same food groups, there are differences in the amounts of each food group recommended. Consistent with this divergence in recommendations, the 2008 NZ Adult Nutrition Survey, being the latest available survey data [13], suggests that the average NZ diet is significantly different from that of the Mediterranean Diet. In particular, the Mediterranean Diet is low in dairy and red meat, which is in contrast to the typically higher meat and dairy intakes evidenced in the NZ Adult Nutrition Survey data. This dietary variation likely reflects New Zealand’s history as an agricultural nation, which has influenced lifestyle practices, most notably in food choice [14].

One study, in a cohort of older adults in New Zealand [15], reported a low percentage of participants adhering to a Mediterranean dietary pattern. Non-Mediterranean populations may encounter a number of challenges in adhering to a Mediterranean dietary pattern. Studies in the US [6,7], Canada [8], and Australia [9] have described difficulties that these populations have with accessing and purchasing particular food groups that are in line with the Mediterranean Diet and adherence to a dietary pattern that is different from habitual intake [16].

One-third of New Zealanders die from cardiovascular diseases annually [17], and approximately 5–6% of the population have Type 2 Diabetes Mellitus (T2DM) [18]. Given the proposed cardiometabolic benefits of a Mediterranean Diet dietary pattern and the potential mismatch between the typical dietary intakes of an agricultural nation such as New Zealand and the Mediterranean Diet, the country-specific modification of public health messaging, policy, and practice may be warranted. At present, we have little understanding of the degree to which the New Zealand population adheres to Mediterranean dietary patterns. The objective of this study was to define the habitual dietary intakes and patterns of a sample of healthy NZ adults and examine the degree to which the New Zealand population’s diet accords with the Mediterranean Diet. To capture the many possible facets of the Mediterranean Diet, we used two approaches: an a priori approach, where a diet quality index of Mediterranean Diet (reference MSDPS) was used to assess the accordance of food and nutrient intake; and an a posteriori approach, which used principal component analysis (PCA) to empirically identify dietary patterns and judge whether they conformed to the Mediterranean Diet.

## 2. Materials and Methods

### 2.1. Study Design and Population

NZ MED was a cross-sectional, web-based study designed to assess current habitual dietary patterns (DPs), macronutrient and micronutrient intakes, and adherence to a Mediterranean Dietary Pattern in healthy New Zealand adults, and it was conducted between June and August 2021. Healthy adults aged 18–70 years living in New Zealand were recruited via social media advertising (utilizing the University of Auckland Facebook and Instagram accounts), professional databases, and email lists. Participants who met the inclusion criteria were provided with a participant information sheet and consent form and gave electronic written informed consent to participate. Ethical approval was obtained from the Auckland Health Research Ethics Committee (Reference: AH22272, expiry 30 April 2024). Participants could reside anywhere in New Zealand, and data collection was managed centrally from Auckland.

The inclusion criteria were healthy adults between 18 and 70 years of age currently residing in NZ. The exclusion criteria were: being unable or unwilling to complete all aspects of the questionnaire; and a diagnosed chronic disease, including but not limited to pre-existing diabetes, cardiovascular disease or related metabolic disease, or any other condition or situation that may affect dietary patterns or diet quality. Final decisions on exclusion were made by the Principal Investigator (TLM).

### 2.2. Questionnaires and Data Collection

The study data were collected and managed using Research Electronic Data Capture (REDCap v. 13.1.32) tools hosted by the University of Auckland. REDCap is a secure, web-based software platform designed to support data capture for research studies [19].

To assess diabetes risk, the AUSDRISK score was used, which consists of ten questions pertaining to simple demographic, lifestyle, and anthropometric information to produce a score which predicts the 5-year risk of T2DM [20]. An AUSDRISK score of five or less is associated with a low risk, scores of six to eleven represent an intermediate risk, and a score of twelve or more represents an intermediate to high risk [20]. Increasing AUSDRISK scores have been shown to be associated with increased weight, body mass index, fasted blood glucose, and metabolic syndrome [21].

### 2.3. Study Variables

Nutrient Intake: Habitual dietary and nutrient intake was estimated with a computerized version of the validated semi-quantitative University of Otago FFQ (short form v.3) [22,23]. Participants were instructed to indicate their usual intake of food items over the past three months, as previously described [22]. Incomplete FFQ were excluded from analysis. The frequency of intake were coded as the average number of servings per week for further analysis. Food items in the FFQ were classified into eight categories based on nutrient similarity, as previously described [22,23]. Total macronutrient and micronutrient intakes were calculated for each participant.

Dietary Patterns: To identify a posteriori dietary patterns, the total frequencies of intake of each of the fourteen food groups were summarized and used as input variables in the dietary patterns analysis. PCA was applied to these food groups to generate various independent dietary patterns (factors) made up of foods with a high degree of inter-correlation. Components were identified based on an Eigenvalue ≥ 1 and the examination of the breakpoint on the Scree plot to determine the number of components that would adequately explain the total variance. The first two principal components were selected for analysis despite other components having an Eigenvalue ≥ 1. The rationale for this was that the first two components exhibited a simple structure and provided the most amount of meaningful data. Varimax rotation was performed to maximize the factor loadings and describe the dietary patterns. Food groups with absolute factor loadings ≥ 0.3 were considered to have a strong association with the dietary patterns. Large positive or negative factor loadings indicated which food groups were important for each component and were used to inform the dietary patterns. Descriptive names were then created based on the food groups with the strongest associations within each pattern (positive loadings > 0.3).

Diet Quality: The Mediterranean-Style Dietary Pattern Score (MSDPS) was developed using the recommended intakes of the thirteen food groups in the Mediterranean Diet food pyramid and assesses dietary quality through adherence to the Mediterranean Diet, as previously described [7,24]. Unique features of this index include accounting for the overconsumption of foods and consumption of foods included and excluded from a Mediterranean dietary pattern. While the MSDPS uses numerical data to measure the frequency of consumption, the FFQ uses categorical data. Therefore, within each item of frequency in the FFQ, the average number of servings was calculated (to the nearest 0.5 servings) for analysis using the MSDPS. Of the thirteen components in the MSDPS, all except ‘Wine’ are relevant for analysis. The FFQ did not provide data pertaining to wine consumption, as it did not make any distinctions between beer, wine, and spirits. This meant the total MSDPS score was out of 120 rather than the previously described 130 [7]. Forty-one FFQ items were found to be relevant for analysis in the MSDPS.

Multiple items from the FFQ were relevant for ‘Fish and other seafood’, ‘Olives, legumes, and nuts’, ‘Potatoes and other starchy roots’, and ‘Sweets’ in the MSDPS. Since the MSDPS scores these items according to the number of servings consumed weekly, the sum of all relevant FFQ items was used to determine the score. Similarly, multiple items from the FFQ were relevant for ‘Wholegrains’, ‘Fruits’, ‘Vegetables’, and ‘Dairy’, where all relevant FFQ items were summed and then divided by seven to determine the score. Additionally, the response to the FFQ item ‘What brand and type of oil do you usually use?’ was used to assign a score for the ‘Olive Oil’ item in the MSDPS. A response including only olive oil would receive a score of ten, a response including olive oil and other vegetable oils would receive a score of five, and a response that did not include olive oil would receive a score of zero. For the items ‘Poultry’, ‘Eggs’, and ‘Meat’, the frequency indicated in each of the respective FFQ items was used to assign a score in the MSDPS [7,24]. The MSDPS is scored continuously as a sum of 13 component scores standardized to a 0–100 point scale and uses a weighting system from 0 to 100% energy contribution of the consumption of foods that are considered part of the Mediterranean Diet. A higher score indicates greater conformity to the Mediterranean Diet [7].

### 2.4. Statistical Analysis

All of the statistical analysis was performed using SPSS Statistics (Version 27. IBM Corp.; Armonk, NY, USA). All statistical tests were two-sided, and *p*-values < 0.05 were considered statistically significant. The characteristics of all the participants who completed the demographics questionnaire were summarized using descriptive statistics. Continuous variables were described as the mean ± standard deviation (SD), and categorical variables were described as the frequency (%). To ensure the suitability of the data for PCA, the Kaiser–Meyer–Olkin (KMO) and Bartlett Tests were performed.

Dietary pattern z-scores were calculated for all respondents to quantify how much their reported intake reflected each dietary pattern relative to the other respondents in the sample. Here, an increasing intake of food groups with positive factor loadings increases the dietary pattern z-score, and an increase in food groups with negative factor loadings decreases the dietary pattern z-score. The dietary pattern and MSDPS scores were categorized into quartiles and mean ± SD values assigned to each quartile. Differences in continuous variables (weight, waist circumference, BMI, energy intake, fiber density, and percentage contribution of macronutrients to energy intake) were compared using ANOVA tests to examine differences across quartiles of dietary pattern scores and MSDPS, adjusted for age. Chi-squared tests were used to determine differences in categorical variables. The variables tested were sex (male/female), % NZ European, and % Māori/Pasifika. Linear regression was used to investigate the association between quartiles of MDSPS (independent variable) and participant characteristics and dietary intake (dependent variables). Potential confounders that were adjusted for in the model were identified as age and the proportion of NZ European participants.

## 3. Results

### 3.1. Population Characteristics

**Figure 1:**
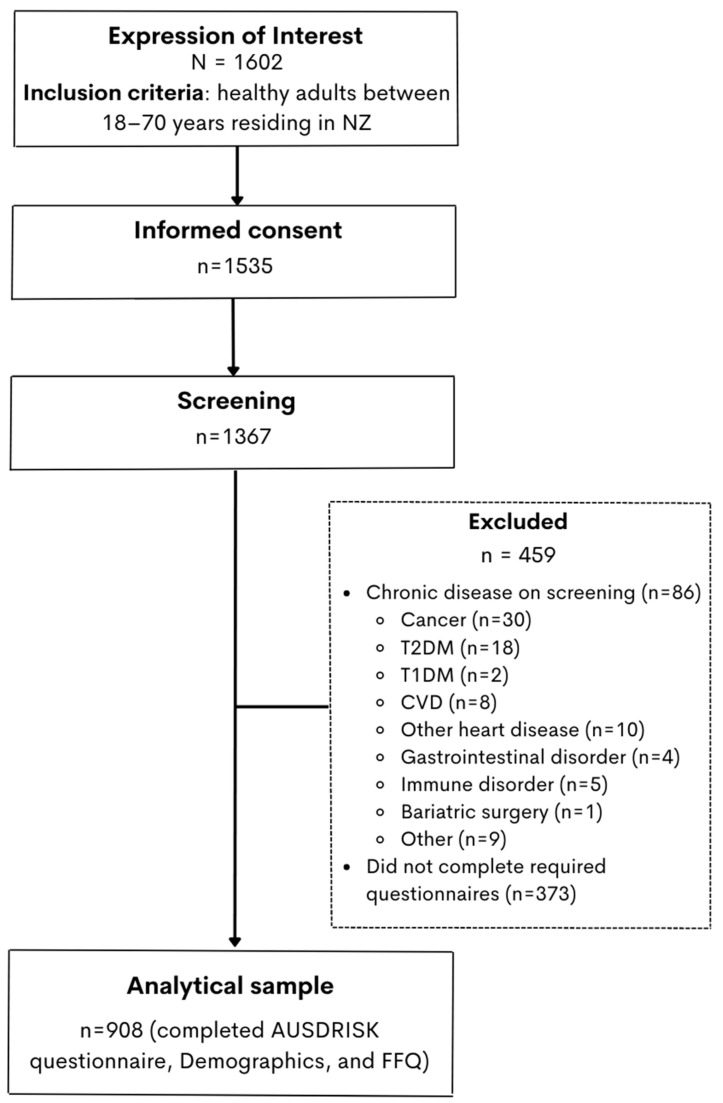
Study participant flow from Expression of Interest to the final Analytical Sample of 908 participants who completed AUSDRISK, Demographics, and Food Frequency Questionnaires.

A total of 1602 individuals expressed interest in participating in NZ MED (Figure 1). Of these, 1535 provided written informed consent. The screening questionnaire was completed by 1367 participants (85% response rate), with 86 participants being excluded following screening due to the presence of a chronic disease. Only those who then completed the AUSDRISK questionnaire [20], the demographics questionnaire, and a New Zealand validated FFQ [22,23] were included in this analysis (*n* = 928).

Participant characteristics are summarized in Table 1. The cohort was predominantly female (88%) and of prioritized NZ European ethnicity (74%). The majority of participants were in the paid employed (74%) and married (64%) categories. The AUSDRISK questionnaire (components not displayed) indicated that the cohort consisted primarily of participants with no family history of diabetes (82%) and no history of high blood glucose (95%) who were not currently being medicated for high blood pressure (91%) and were not current smokers (98%). Most of the cohort consumed fruit and/or vegetables every day (94%) and engaged in 2.5 h or less of physical activity per week (76%). Most participants (51%) were categorized as ‘high’ according to their AUSDRISK score.

### 3.2. Nutrient Intakes

Participants’ mean ± SD energy and macronutrient and micronutrient intakes are summarized in Table 2. After excluding outliers (*n* = 15), 893 participants’ data were included. The percent contributions of the macronutrients to energy intake were compared to the Acceptable Macronutrient Distribution Ranges (AMDR) [25]. Notably, the mean total fat intake exceeded the upper end of the AMDR (25–35% energy intake) by 11%, and the mean ± SD saturated fat intake exceeded the recommended <10% energy intake by 50%.

### 3.3. Dietary Patterns

**Figure 2:**
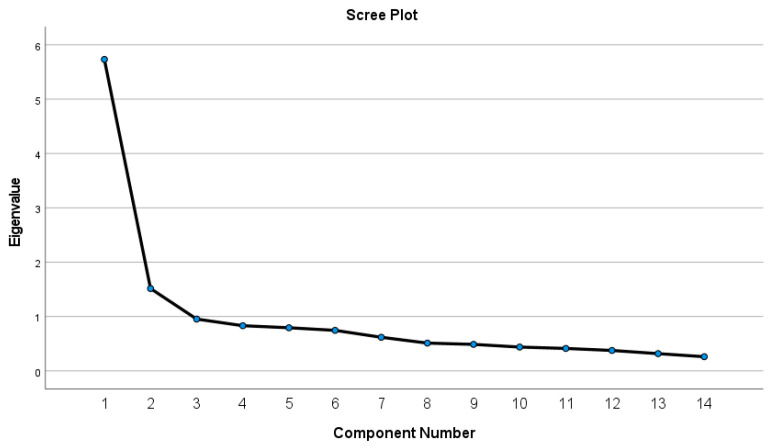
Scree plot for the identification of dietary patterns by principal component analysis.

Two dietary patterns for the whole cohort were identified (Figure 2), accounting for 52% of the variability in the sample (41% dietary pattern 1 and 11% dietary pattern 2). The first dietary pattern, labeled ‘Discretionary’, was characterized by positive loadings on processed meat, meat/poultry, fast food, sweet drinks, and sugar/sweets/baked goods. The second dietary pattern, labeled ‘Guideline’, was characterized by positive loadings on vegetables, fruits, and eggs/beans and positive associations with wholegrains and oil/spreads (Table 3).

Demographics, medical history, and nutrient intake were considered in relation to dietary pattern z-scores to identify any differences between those with higher adherence to the ‘Discretionary’ dietary pattern and ‘Guideline’ dietary pattern. Quartile one (Q1) represents the least adherence, and quartile four (Q4) represents the most adherence (Table 4). Across the quartiles of ‘Discretionary’ dietary pattern z-scores, those more adherent (Q4) were younger, were more likely to be of Māori/Pacific ethnicity, and had a lower proportion of female participants. The ‘Discretionary’ dietary pattern included more energy-dense foods, with those more adherent (Q4) having a higher intake of energy, higher percent contribution to energy from total and saturated fat, lower fiber density, and higher BMI than those who were the least adherent (Q1) to the dietary pattern. AUSRISK scores were similar across all ‘Discretionary’ quartiles. Across quartiles of ‘Guideline’ dietary pattern z-scores, those who were more adherent (Q4) were older, a greater proportion of them were female, they were of NZ European ethnicity, and they had higher energy intakes and fiber densities compared to those who were the least adherent (Q1). Carbohydrates contributed a higher percent to the total energy intake in those who were more adherent to the dietary pattern. For nutrients of interest in a Mediterranean dietary pattern such as unsaturated fatty acids, the percent energy contribution of MUFAs in the ‘Guideline’ dietary pattern decreased significantly across quartiles as the percent energy contributions of PUFAs increased significantly across quartiles. The inverse was seen across the ‘Discretionary’ dietary pattern quartiles.

### 3.4. Diet Quality

The mean ± SD MSDPS score for the total cohort was 20.9 ± 7.4, with an average weighting score of 0.53 ± 0.12. MSDPS scores were split into quartiles, and the distributions of potential confounding variables were examined across these. MSDPS scores had means ± SD of 12 ± 2.8, 18 ± 1.4, 23 ± 1.5, and 30.5 ± 4.4 across quartiles, with higher scores being associated with older age and NZ European ethnicity (Table 5). There were no differences between any variables of medical history and according to having completed higher education, but there was a significant difference (*p* < 0.001) in the proportion of participants who had a high risk of developing T2DM according to AUSDRISK. Participants in Q1 of the MSDPS score, meaning they were the least adherent to a Mediterranean Diet, had higher energy and total fat intakes and lower protein and carbohydrate intakes compared to those in Q4 (most adherent to a Mediterranean Diet within the sample).

## 4. Discussion

In this sample of New Zealand adults, two principal DP (named ‘Discretionary’ and ‘Guideline’) explained 52% (41% and 11%, respectively) of the variance in specific nutrients, namely, energy, fiber density, MUFA and PUFA, of which all excluding energy are cornerstones of the Mediterranean Diet. There was low adherence to the Mediterranean Diet among NZ adults overall (mean MSDPS score of 20.9 out of a possible 100). However, in relation to T2DM risk, AUSDRISK scores increased across quartiles of MSDPS scores, likely driven by participant age.

Dietary patterns identified in the 2008 NZ ANS included a ‘Healthy’ DP, which was characterized by breakfast cereal, low-fat milk, soups, yoghurt, fruit, tea, pies/pastries, potato chips, white bread, takeaway foods, sugar-sweetened beverages, and alcoholic beverages, and a ‘Traditional’ DP, which was characterized by beef, vegetables, starchy vegetables, milk and cream, sugar, tea, and coffee and was low in takeaway foods [1]. Neither DP identified in the present study resembles those described in the 2008 NZ ANS [1]. There was a mixture of positive loadings for food groups that fall under the ‘Healthy’ and ‘Traditional’ classifications in the ‘Discretionary’ and ‘Guideline’ DP. These differences are likely a reflection of changes in typical food choices and habits across time, with a shift away from traditional agricultural Anglo-Saxon choices since 2008. In addition, there were differences in the populations represented in the 2008 nutrition survey and the current dataset.

The current investigation was conducted from 2020 to 2021, during the height of the COVID-19 pandemic and the associated lockdown or quarantine procedures, which was likely to have impacted dietary behaviors. While it is not possible to quantify these changes using the FFQ, a consideration of how the pandemic has altered dietary behaviors over the long term deserves further investigation. A recent publication on dietary intakes during the COVID-19 pandemic response in NZ demonstrated an “overall shift toward an unhealthy eating pattern” with an increased consumption of sweet snacks (41%), salty snacks (33%), and sugary drinks (20%) during the lockdown, with those aged under 50 years more likely to report adverse changes to dietary habits [26] and a notable increase in the use of ‘comforting’ recipes during the lockdown, which was associated with higher scoring in the ‘Unhealthy DP’ [27]. Both studies reported an overall decrease in the nutritional quality of diets during the COVID-19 pandemic [26,27], which may explain, to some degree, the relatively low adherence to the NZ Dietary Guidelines found in this study.

Adherence to the ‘Discretionary’ DP had significant associations with several demographic factors. The proportion of females decreased with an increasing adherence to the ‘Discretionary DP’, indicating that males tend to consume a more ‘Discretionary’ DP. These sex differences have previously been reported, where women eat more fruits, vegetables, and wholegrain products than men [28,29]. This trend was also reported in the 2008 ANS, where females were more likely to be associated with the ‘healthy’ DP than males [30]. Conversely, the consumption of red meat, alcohol, high-sugar foods, and various high-starch foods such as potatoes and bread is higher in males and attributed mainly to psychological and socio-cultural factors such as general health and nutrition awareness, body image (often heavily shaped by societal expectations), and dieting behaviors [28,29]. Age was also significantly associated with adherence. The average age in Q4 (highest adherence to the Discretionary DP) was 43 years, and in Q1 (lowest adherence), it was 51 years, as previously reported in the literature [31,32]. The uneven distribution of females versus males in this cohort is acknowledged and may skew our findings; however, understanding the underlying factors that drive and sustain eating behaviors and patterns (and any potential differences between sexes and age groups) may help inform interventions and public health messages that seek to spark long-term dietary change in the direction of the Mediterranean Diet.

A significant association was also seen between the ‘Discretionary’ DP and ethnicity. Higher adherence was associated with being Māori/Pacific, while lower adherence was associated with being NZ European. These findings are echoed across other NZ research, including the EXPLORE study, where Māori and Pacific women were more likely to follow the ‘refined and processed’ DP compared to NZ European women [31], Growing Up in New Zealand, where the ‘Junk’ DP was found to be significantly associated with being Māori/Pacific, and the 2008 NZ ANS, which reported that Māori/Pacific women were three times more likely to eat ‘fast food’ three or more times per week than women who were not of Māori or Pacific Island descent [13,31]. While the NZ MED cohort was predominantly female and of NZ European ethnicity, direct comparisons with these samples cannot be made. However, the similarities may reflect the well-documented differences in underlying risk factors between ethnicities, such as the socioeconomic status, which mediates diet-related disease through differential access to food, education, employment, and healthcare [33,34]. Since diet-related non-communicable diseases disproportionately affect Māori and Pacific peoples in New Zealand, the particular focus must be placed on culturally responsive dietary interventions [35,36,37].

The findings from this study support the observation that the caloric intake in individuals is relatively stable across DPs, and dietary change is characterized by substitution effects where high intakes of some foods are counteracted by lower intakes of other foods [38]. We found that the ‘Guideline’ DP had higher intakes of PUFA, whereas the ‘Discretionary’ DP had higher intakes of SFA. While PUFA and SFA contribute the same energy per gram, they have different impacts on future metabolic disease risk [13,31,39,40,41]. Replacing dietary SFA with PUFA has been reported to lower coronary heart disease events and improve glycemic control, insulin resistance, and insulin secretion capacity [13,31,39,40,41]. This reinforces the importance of analyzing DPs rather than single nutrients, as DPs capture the interrelatedness of food choices and the effect of cumulative exposure on health outcomes [38]. Those with higher adherence to the ‘Guideline’ DP had higher intakes of vegetables, eggs/beans, fruits, oil/spreads, whole grains, and coffee/tea. The ‘Guideline’ DP showed significant associations with age, sex, and ethnicity. As expected, these associations were opposite to the ‘Discretionary’ DP. Moreover, despite the known link between excessive energy intake and obesity (and, consequently, obesity-related disease), absolute energy or macronutrient intake is not a focus of the Mediterranean Diet. Instead, the Mediterranean Diet emphasizes the food sources [42]. A characteristic of the Mediterranean Diet emphasizes plant sources of proteins such as legumes over animal sources [12,24].

The average weighting score of 0.53 indicates that only approximately half of the food sources contributing to the diet of NZ adults is comprised of foods that are aligned with the Mediterranean Diet. This suggests that the current New Zealand diet is considerably different than the Mediterranean Diet, and future analysis of the contribution of individual foods to the total MSDPS score is recommended, as both Mediterranean and non-Mediterranean foods were present within the 14 food groups identified in the DP analysis. Ultimately, given that the Mediterranean dietary pattern has been associated with a lower risk of cardiometabolic diseases, this suggests that there is room to shift the New Zealand diet towards a Mediterranean pattern as a means of effecting a positive impact on cardiovascular disease and diabetes risk. The greatest challenge will be supporting the adoption of and long-term adherence to such a dietary pattern.

Adherence to either dietary pattern (discretionary and guideline) was not associated with the AUSDRISK score, a validated type 2 diabetes risk questionnaire [20]. One possible reason is that the AUSDRISK questionnaire is weighted towards non-modifiable risk factors (age, gender, ethnicity/country of birth, and family history of T2DM), meaning that it may not be the best tool for capturing dietary and other modifiable risk factor contribution to type 2 diabetes risk, which may be smaller than non-modifiable. On average, those in the high-risk group were older, and this group had a significantly higher percentage of Māori and Pacific participants and participants with a family history of T2DM. However, there were no significant differences in the percentage of participants who ate fruit/vegetables daily between the two groups. This suggests that participants categorized as high-risk may be placed in this category primarily due to non-modifiable risk factors, despite similar dietary habits to those in the low-intermediate risk group. The association between increasing age and higher MSDPS is likely confounded by the influence of age on the AUSDRISK score. Of the remaining modifiable risk factors (history of high BGL, current medication for high BP, smoking status, fruit/vegetable consumption, physical activity, and waist circumference), four can be expected to increase with age; as metabolic disease develops slowly over time, an older person is more likely to have a history of high BGL, be medicated for high BP, be less active, and have a higher waist circumference. As those in the high-risk group were found to be significantly older, some participants in the low-intermediate-risk group may represent those before disease development. Interestingly, while waist circumference was not significantly different between the two groups, BMI was significantly higher in the high-risk group. On average, those in the low-intermediate-risk group had a healthy BMI (24.9 kg/m^2^), while those in the high-risk group were overweight (27.6 kg/m^2^). This is what we would expect, as obesity is linked to the development of T2DM [21].

### 4.1. Limitations

A few methodological issues merit comment. The study had a large sample size and used appropriate sampling methods, but the results cannot be generalized, as our population was predominantly female (88%), was of NZ European ethnicity (74%), and had completed some form of higher education; therefore, it is not fully representative of the NZ population, and caution is recommended when interpreting these findings. This research was also conducted during the COVID19 pandemic, which has been documented to have affected the nutritional quality of dietary intakes [26,27]. Dietary intake was measured using a population-validated FFQ but was based on self-reported data and is subject to recall bias. FFQ have their limitations; however, the Otago short-form FFQ has been demonstrated to show good reproducibility and validity when ranking individuals by their nutrient intakes [22]. This FFQ is widely used in NZ research and has been reported to be a time-efficient way to assess the nutrient intakes of NZ adults [22,23]. There are several different indices for assessing the Mediterranean Diet, so, the results on Mediterranean Diet adherence could vary with the index used. However, MSDPS has been shown to assess adherence accurately and concordantly with other indices [43]. The cross-sectional design did not allow us to examine the prospective influence of socio-demographic factors, lifestyles, and other variables. PCA factor analysis is arbitrary, such as categorization into food groups, the number of factors to extract, the method of rotation, and the labeling of the factors.

### 4.2. Implications for Future Research

Follow-up studies need to understand the impact of the Mediterranean Diet on cardiometabolic health in NZ and the barriers and facilitators of adopting it. Future research might consider targeting dietary interventions according to demographic factors such as age and sex as opposed to (or in conjunction with) the level of disease risk [44]. In the absence of an updated NZ ANS, it remains a priority to recruit a representative sample to capture the diverse subpopulations residing in NZ. The consideration of factors such as these is not only pertinent for cultural safety but may also impact the effectiveness of public health interventions [45]. Cultural safety and equity continue to be an important part of the dialogue in designing and implementing population-level programs [44]. This will be crucial in developing an equitable and efficacious dietary intervention.

## 5. Conclusions

This study has provided evidence that the current dietary habits of NZ adults can be depicted as two distinct DPs. Adherence to both DPs was significantly associated with age, sex, and ethnicity, adding to the existing evidence that these factors are significant predictors of dietary habits. The diet quality defined by MSDPS was relatively poor, indicating that a significant shift in food choices will be required if the Mediterranean Diet is to be adopted in an NZ context. This preliminary study will inform further research on dietary interventions in NZ adults and the development of equitable and efficacious interventions for promoting metabolic health in Aotearoa NZ.

## Figures and Tables

**Table 1:** Characteristics and demographics of the analytical sample ^1^ (*n* = 908).

Characteristics	Total (*%*), or Mean ± SD
*N*	908
Sex	
Female	794 (88)
Male	114 (13)
Age (years)	48 ± 17
Ethnicity	
NZ European	675 (74)
Māori	25 (3)
Pacific	12 (1)
Asian	71 (8)
Other European	101 (11)
Other	24 (3)
Employment status	
Paid employee/employer	663 (73)
Not employed and starting a new job in 4 weeks	7 (1)
Not employed	176 (19)
Other (retired)	47 (5)
Relationship status	
Married	584 (64)
Single/never married	218 (24)
Divorced/separated	86 (10)
Widowed	20 (2)
Highest secondary school qualification	
No secondary school qualification	13 (1)
National Certificate Level 1, 2, or 3	724 (80)
Overseas secondary education	171 (19)
Highest qualification	
National Certificate Level 1–4/Trade Certificate	49 (5)
University	494 (54)
Post-Graduate study, including PhD	217 (24)
Other	25 (3)
Missing	123 (14)
Weight (kg)	72 ± 16
BMI (kg/m^2^)	26 ± 11
Waist circumference (cm)	83 ± 17
Total AUSDRISK score	11 ± 4
AUSDRISK category	
Low	27 (3)
Intermediate	419 (46)
High	462 (51)
MSDPS score	20.9 ± 7.4

^1^ The analytical sample includes those who completed the AUSDRISK, demographics, and FFQ. Abbreviations: AUSDRISK, Australian Type 2 Diabetes Risk Assessment Tool; BMI, Body Mass Index; MSDPS, Mediterranean Dietary Pattern Score.

**Table 2:** Nutrient intake and percent contribution to energy intake by macronutrients for the analytical sample (*n* = 893).

Nutrient		Mean ± SD
Energy (kJ)		7277.1 ± 2103.5
Carbohydrate (g)		180.1 ± 67.5
Protein (g)		77.0 ± 26.3
Total fat (g)		75.5 ± 26.5
Saturated fat (g)		38.1 ± 16.5
MUFA (g)		25.9 ± 9.2
PUFA (g)		21.4 ± 8.6
Cholesterol (mg)		228.5 ± 150.5
Fiber (g)		29.6 ± 12.4
Calcium (mg)		795.9 ± 382.9
Iron (mg)		12.5 ± 4.4
Zinc (mg)		10.6± 3.5
Folate (µg)		456.4 ± 181.7
Vitamin C (mg)		142.1 ± 76.6
Vitamin D (µg)		2.3 ± 1.9
**Contribution to Energy Intake (% Energy Intake)**		
	**AMDR**	**Mean ± SD**
Carbohydrate (%)	45–65% EI	41.2 ± 9.8
Protein (%)	15–25% EI	17.9 ± 3.9
Total fat (%)	20–35% EI	39.1 ± 8.1
Saturated fat (%)	<10% EI	19.8 ± 6.1
MUFA (%)		13.5 ± 3.1
PUFA (%)		11.0 ± 2.9

Abbreviations: AMDR, Acceptable Macronutrient Distribution Range; EI, energy intake; MUFA, monounsaturated fatty acids; PUFA, polyunsaturated fatty acids.

**Table 3:** Dietary patterns derived from principal component analysis of food groups using rotated component matrices in the analytical sample (*n* = 893).

Food Group	Discretionary	Guideline
Processed meat	**0.810**	0.054
Meat, poultry	**0.770**	0.166
Fast food	**0.548**	−0.099
Sweet drinks	**0.474**	−0.076
Sugar, sweets, baked goods	**0.302**	−0.234
Vegetables	−0.030	**0.823**
Eggs, beans	0.082	**0.748**
Fruits	−0.032	**0.463**
Oil, spreads	−0.028	0.272
Wholegrains	−0.219	0.213
Dairy foods	0.212	−0.081
Coffee, tea	−0.203	0.093
Starches	0.101	0.096
Alcoholic beverages	0.008	0.072

Bold values represent loading ≥ 0.30 to aid the labeling of dietary patterns.

**Table 4:** Demographic and health information across quartiles of PCA-derived z-scores of DPs in the analytical sample (*n* = 908).

	Discretionary					Guideline				
	Q1 (*n* = 227)	Q2 (*n* = 227)	Q3 (*n* = 227)	Q4 (*n* = 227)	*p* Value	Q1 (*n* = 227)	Q2 (*n* = 227)	Q3 (*n* = 227)	Q4 (*n* = 227)	*p* Value
**Demographics**										
Age (years)	51 ± 16	51 ± 15	48 ± 15	43 ± 16	**<0.001** ^a^	41 ± 15	48 ± 17	51 ± 15	53 ± 14	**<0.001** ^a^
Sex (% female)	220 (94.0%)	213 (91.0%)	202 (86.7%)	181 (78.0%)	**<0.001** ^b^	191 (82.0%)	209 (90.5%)	204 (87.6%)	212 (89.8%)	**0.023** ^b^
NZ European (%)	178 (76.1%)	184 (78.0%)	173 (73.6%)	150 (64.4%)	**0.005** ^b^	139 (60.2%)	193 (81.4%)	175 (74.5%)	178 (75.7%)	**<0.001** ^b^
Māori/Pacific (%)	5 (2.1%)	11 (4.7%)	11 (4.7%)	25 (10.7%)	**<0.001** ^b^	20 (8.7%)	10 (4.2%)	15 (6.4%)	7 (3.0%)	**0.039** ^b^
Higher Education (% yes)	193 (85)	191 (84)	202 (89)	199 (88)	0.397 ^b^	191 (84)	206 (91)	199 (88)	189 (83)	0.076 ^b^
**Medical history**										
Weight (kg)	68 ± 13	72 ± 15	72 ± 15	76 ± 20	**<0.001** ^a^	74 ± 19	71 ± 14	72 ± 16	71 ± 16	0.166 ^a^
Waist circumference (cm)	80 ± 15	84 ± 16	84 ± 16	84 ± 20	**<0.001** ^a^	84 ± 19	82 ± 16	83 ± 17	84 ± 16	0.751 ^a^
BMI (kg/m^2^)	24.6 ± 4.5	27.6 ± 20.0	25.9 ± 5.0	27.1 ± 6.6	**0.007** ^a^	26.2 ± 6.0	26.0 ± 12.4	26.3 ± 5.9	26.6 ± 16.3	0.957 ^a^
AUSDRISK score	11 ± 4	11 ± 4	11 ± 4	11 ± 4	0.392	10 ± 4	11 ± 4	11 ± 4	12 ± 4	**<0.001**
**Dietary intake**										
Energy intake (kJ/day)	7275.2 ± 2192.3	6728.2 ± 2047.9	6997.5 ± 1912.9	8645.7 ± 3242.7	**<0.001** ^a^	6110.4 ± 2730.1	6751.7 ± 2356.5	7584.2 ± 1811.1	9190.6 ± 1941.0	**<0.001** ^a^
Fiber density (g/kJ)	5.1 ± 1.2	4.1 ± 0.9	3.7 ± 0.9	3.2 ± 0.9	**<0.001** ^a^	3.4 ± 1.0	3.9 ± 1.0	4.2 ± 1.1	4.7 ± 1.3	**<0.001** ^a^
Carbohydrate (%)	42.4 ± 10.2	40.7 ± 9.7	40.5 ± 9.4	41.2 ± 9.8	0.093 ^a^	40.5 ± 10.4	40.5 ± 8.9	40.7 ± 10.1	43.0 ± 9.5	**0.010** ^a^
Protein (%)	17.3 ± 3.7	18.0 ± 4.0	17.9 ± 3.8	18.4 ± 4.1	**0.003** ^a^	17.9 ± 4.3	17.9 ± 3.7	17.9 ± 3.7	17.8 ± 3.9	0.953 ^a^
Total fat (%)	38.2 ± 8.3	39.0 ± 8.3	39.6 ± 8.0	40.7 ± 7.4	**0.003** ^a^	39.3 ± 8.2	39.9 ± 7.6	39.7 ± 8.3	38.6 ± 8.1	0.309 ^a^
Saturated fat (%)	18.3 ± 5.0	19.5 ± 5.6	20.0 ± 6.2	21.7 ± 7.0	**<0.001** ^a^	20.7 ± 7.5	19.7 ± 6.1	19.4 ± 5.4	19.6 ± 5.3	0.099 ^a^
MUFA (%)	12.3 ± 3.3	13.2 ± 2.8	13.8 ± 3.1	14.7 ± 3.0	**<0.001** ^a^	14.2 ± 3.4	13.7 ± 3.0	13.5 ± 3.2	12.7 ± 3.0	**<0.001** ^a^
PUFA (%)	11.4 ± 3.2	11.3 ± 3.0	10.9 ± 2.6	10.8 ± 2.7	**0.026** ^a^	10.6 ± 2.7	11.2 ± 2.9	11.4 ± 2.8	11.2 ± 3.0	**0.015** ^a^

Data are presented as the count (%) or mean ± s.d. Abbreviations: BMI, body mass index; MUFA, monounsaturated fatty acids; NZ, New Zealand; PUFA, polyunsaturated fatty acids. ^a^ One-way ANOVA, ^b^ Chi Square test. Significant results (*p* < 0.05) are highlighted in bold.

**Table 5:** Demographic and health information across quartiles MSDPS scores in the analytical sample (*n* = 908).

	MSDPS				
	Q1 (*n* = 227)	Q2 (*n* = 228)	Q3 (*n* = 225)	Q4 (*n* = 228)	*p* Value
**MSDPS score**	12.0 ± 2.8	18.0 ± 1.4	23.1 ± 1.5	30.5 ± 4.4	**<0.001**
**Demographics**					
Age (years)	42 ± 16	47 ± 16	51 ± 15	53 ± 15	**<0.001** ^b^
Sex (% female)	194 (82.9)	209 (89.3%)	202 (88.2%)	211 (89.4%)	0.107 ^b^
NZ European (%)	144 (61.8%)	180 (76.3%)	176 (75.5%)	185 (78.4%)	**<0.001** ^b^
Māori/Pacific (%)	19 (8.2%)	8 (3.4%)	13 (5.6%)	12 (5.1%)	0.156 ^b^
Higher Education (% yes)	188 (83)	196 (86)	200 (89)	201 (88)	0.230 ^b^
**Medical history**					
Weight (kg)	71 ± 18	71 ± 15	73 ± 18	73 ± 14	0.869 ^a^
Waist circumference (cm)	81 ± 19.0	83 ± 17	85 ± 16	84 ± 15	0.675 ^a^
BMI (kg/m^2^)	25.6 ± 6.20	25.6 ± 5.5	26.9 ± 12.8	27.0 ± 16.1	0.780 ^a^
AUSDRISK score	10 ± 4	11 ± 4	12 ± 4	12 ± 4	**<0.001** ^a^
**Dietary intake**					
Energy intake (kJ/day)	7847.0 ± 3333.9	7704.0 ± 2331.0	7039.0 ± 2216.4	7057.4 ± 1836.1	**0.001** ^a^
Fiber density (g/kJ)	4.027 ± 1.518	3.964 ± 1.135	3.899 ± 1.012	4.271 ± 1.025	**0.008** ^a^
Carbohydrate (%)	40.1 ± 11.0	41.2 ± 9.1	41.2 ± 9.3	42.2 ± 9.5	**0.013** ^a^
Protein (%)	16.6 ± 4.0	17.4 ± 3.9	18.4 ± 3.6	19.1 ± 3.6	**<0.001** ^a^
Total fat (%)	40.3 ± 9.0	39.8 ± 7.6	40.1 ± 7.7	37.3 ± 7.4	**<0.001** ^a^
Saturated fat (%)	19.1 ± 7.9	20.1 ± 5.6	20.6 ± 19.6	19.6 ± 4.9	0.107 ^a^
MUFA (%)	13.6 ± 3.6	13.7 ± 3.2	13.8 ± 2.9	13.0 ± 2.8	**0.007** ^a^
PUFA (%)	12.0 ± 3.5	11.3 ± 2.6	10.9 ± 2.5	10.2 ± 2.5	**<0.001** ^a^

Data are presented as the count (%) or mean ± s.d. Abbreviations: BMI, body mass index; MUFA, monounsaturated fatty acids; NZ, New Zealand; PUFA, polyunsaturated fatty acids; Q, quartile. ^a^ One-way ANOVA, ^b^ Chi Square test. Significant results (*p* < 0.05) are highlighted in bold.

## Data Availability

The data presented in this study are available on request from the corresponding author.
